# Conformational Mobility of GOx Coenzyme Complex on Single-Wall Carbon Nanotubes

**DOI:** 10.3390/s8128453

**Published:** 2008-12-18

**Authors:** Feng Liu, Xue-song Ye, Tao Wu, Chang-Tao Wang, Jia-wei Shen, Yu Kang

**Affiliations:** 1 Collage of Biomedical Engineering & Instrument Science, University of Zhejiang, P.R. China. EMail: fengliu@mail.bme.zju.edu.cn; 2 Key lab of Biomedical Engineering of Ministry of Education, University of Zhejiang, P.R. China.; 3 Department of Chemistry, University of Zhejiang, P.R. China. E-Mails: poopwill@vip.sina.com; kiddsuper@hotmail.com

**Keywords:** Molecular mechanism, Immobilization, Conformational mobility, Embedded coenzyme

## Abstract

A critical issue in bioelectrochemical applications that use electrodes modified by Single Wall Carbon Nanotubes (SWCNTs) is to ensure high activity of the catalytic site of an immobilized enzyme protein interacting with nanomaterials. Since Flavin Adenine Dinucleotide (FAD), a coenzyme of glucose oxidase (GOx), is the active center of the catalytic site, conformation of which could determine the activity of enzyme, it is important to understand the dynamic mechanism of its conformational mobility while GOx is adsorbed on SWCNTs with multiple orientations. However, this dynamic mechanism still remains unclear at the atomic level due to the coenzyme being embedded in the apo-GOx and the limitations of appropriate experimental methods. In this study, a molecular dynamics (MD) simulation was performed to investigate the conformational mobility mechanism of the coenzyme. The trajectory and the interaction energy clearly indicate that the adsorption of GOx onto SWCNTs plays an important role in the conformational mobility of the coenzyme, and its mobility is greatly affected by the distribution of water molecules due to it being hydrophobic.

## 1. Introduction

Glucose oxidase (GOx) electrodes have been extensively studied as a foundation for constructing biosensors, biomedical devices, enzymatic bioreactors and biofuel cells [1-6]. The most critical challenge in these applications is to immobilize the GOx so that it retains its enzymatic activity and permits fast and efficient electron transfer from the catalytic center to the electrode [7-10]. In order to achieve this, suitable electrode materials such as nanomaterials and special techniques for immobilizing enzymes on the electrode surface have been developed. Carbon nanotubes were first introduced by Iijima in 1991 [11] and they have come to be regarded as being a very attractive nanomaterial for a wide range of applications [5-6, 12]. Many experiments have been carried out to exploit the unique properties of SWCNTs that can lead to the preservation of catalytic activity and to the achievement of direct electron transfer with the redox active center of the adsorbed oxidoreductase in SWCNT-modified electrodes [5, 13-14]. It had been found that GOx complex and FAD coenzymes can spontaneously adsorb onto annealed carbon nanotubes with an armchair chirality to improve their bioelectrochemical performance respectively while these complexes are cast onto glassy carbon electrodes (GCEs) [5, 13-14]. Moreover, in a cyclic voltammogram experiment, the peak current of intact GOx on a SWCNT-modified GCE was found to be almost 10 times greater than that on an unmodified GCE, but still less than that of the electroactive FAD directly on SWCNT-modified GCE [13]. At one time, Wohlfahrt *et al*. reported that Glu412 bound to His559 was capable of modulating powerfully its catalytic activity by affecting all the rate constants in the reductive and the oxidative half-reaction of the catalytic cycle while those amino acid residues of apo-GOx along with FAD are in the active site of enzyme [29]. From these cases, it can be apparent that the conformation of FAD in apo-GOx could determine the activity of intact enzyme by influencing on the active structure of redox site. Consequently, in order to recognize what are about the change of activity with GOx complex adsorbed on SWCNTs, it would be worthy of exploring the conformational mobility mechanism of FAD coenzyme while GOx complex is non-covalently adsorbed on SWCNTs with multiple orientations. Of cause, those experiments on SWCNT-modified electrodes have proven to be an essential experimental base for gaining a fundamental understanding of biological redox reactions and for evaluating potential denaturation mechanisms due to the interaction with SWCNTs [13-14]. However, the dynamic mechanism of these phenomena and processes at atom level still remains somewhat unclear due to the FAD coenzyme embedded in the apo-GOx and the limitations of appropriate experimental methods including AFM and STM.

Recently, MD simulations have been demonstrated to minimize unnecessary costs and the need to perform complicated experiments, and can provide a convenient and excellent semi-theoretical platform for estimating broad interactions between biomolecules and inorganic materials on the atomic level [15-20]. In this study, an MD simulation with multiple adsorption orientations of the protein was conducted to investigate the dynamic mechanism of the conformational mobility of a FAD coenzyme under the interaction between intact GOx and the sidewall of a metallic SWCNT. This investigation is based on previous research performed by our group [16-19], and could help us to make further clear some critical issues about the immobilization of enzyme with SWCNTs in bioelectrochemical applications.

## 2. Results and Discussion

### 2.1. The conformational change of FAD

**Figure 1.**
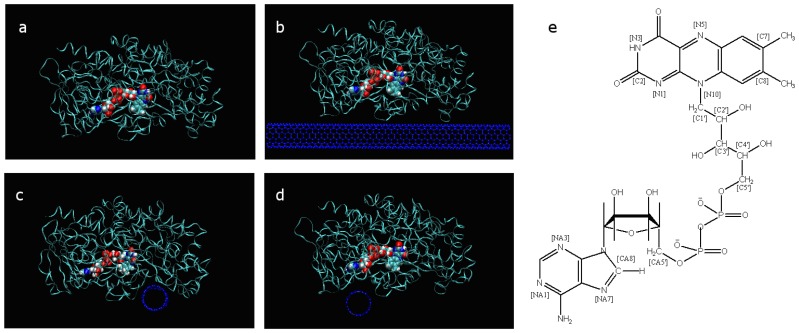
(a) System A with a water box size of 99.5×69.5×79.7 Å^3^; (b) system B with a water box size of 124.0×91.6×82.5 Å^3^, in which SWCNT covers two pockets; (c) system C with a water box size of 124.1×88.0×98.0 Å^3^, in which SWCNT is close to the primary pocket; (d) system D with a water box size of 122.7×92.0×101.9 Å^3^, in which SWCNT is close to the secondary pocket. In these figures, water molecules are not displayed for clarity. (e) The chemical structural of formula of FAD tagged with an atom identifier.

**Figure 2.**
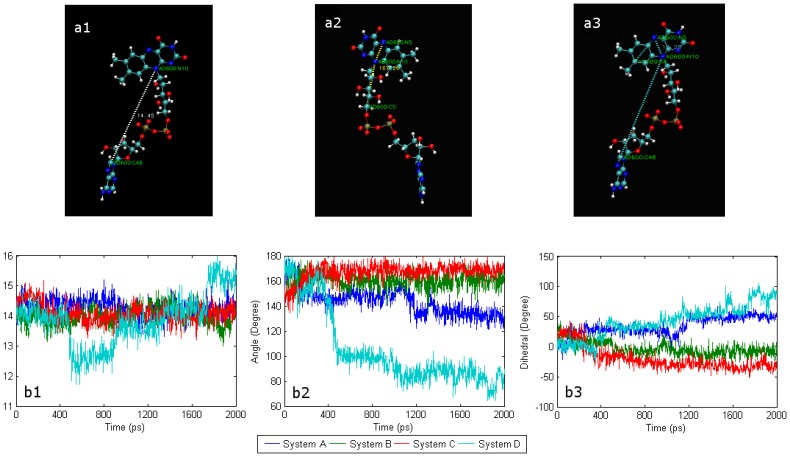
(a1) Distance (N10-CA8) represents the distance between the isoalloxazine and the adenine of FAD; (a2) Angle (N5-N10-C5′) represents the bending deflection of the virtual axis (N5-N10) of isoalloxazine relative to the virtual axis (N10-C5′) of the ribitol segment; (a3) Dihedral angle (C8-N5-N10-CA8) reflects the degree of rotation of the isoalloxazine plane about the virtual axis (N5=N10); (b1) Trends for the distance (C8-CA8), (b2) trends for the angle (N5-N10-C5′) and (b3) trends for the dihedral angles (C8-N5-N10-CA8).

Despite being tightly wrapped in apo-GOx by non-bonded interaction forces that include vdW forces and the electrostatic interaction, FAD still exhibits great mobility in the tunnel of the apo-GOx. A number of structural parameters, including distances, angles and dihedrals, were introduced in this study to describe the fine structural features and evaluate the mobility of FAD. The atom tags of FAD and its formula are shown in Figure 1, and the above-mentioned parameters are depicted in Figure 2a.

In an aqueous solution, FAD that has a large bending deflection can gradually return to a certain extension on its own accord [22]. By analyzing the molecular trajectories, the mobility of FAD in system D was found to be distinctly different from that in system A, being strongly affected by the presence of a SWCNT. Figure 2a illustrates that the distance (N10-CA8) fluctuates more in system D during the 2-ns simulation than those in the other three systems. In contrast with system A, the distance (N10-CA8) is still less than 2 Å in system D at the end of the 2-ns MD simulation. The fluctuations of this distance in systems B and C are similar to that in system A. As shown in Figure 2b, the trend for the angle (N5-N10-C5′) in system B is very similar to that for system C; whereas it deviates somewhat from that for system A, and greatly from than that of system D. Figure 2c shows that the trend of the dihedral angle (C8-N5-N10-CA8) of system B is very similar with system C, but differs greatly from that of system D. At the same time, it is apparent that the trend in the change of system A is somewhat similar to that for systems A and B.

### 2.2. The interaction of FAD with SWCNT and apo-GOx

**Figure 3.**
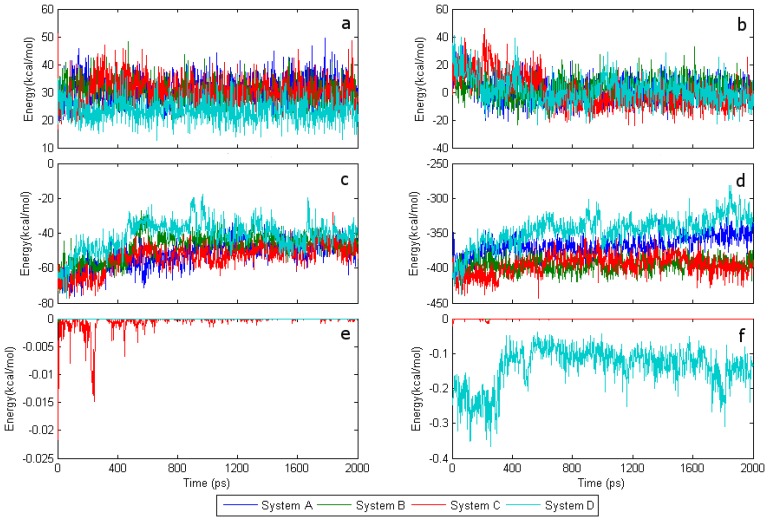
Energy trends during 2-ns MD simulation. (a) Potential energy of isoalloxazine; (b) potential energy of FAD; (c) interaction energy between isoalloxazine and apo-GOx; (d) interaction energy between FAD and apo-GOx; (e) interaction energy between isoalloxazine and SWCNT; (f) interaction energy between FAD and SWCNT.

Since the FAD coenzyme embeds in apo-GOx, in order to find the critical factors that affect the conformational fluctuations, the interaction energy between apo-GOx and the SWCNT should be investigated together with the potential energy of FAD. The intensity of the vdW force at the interaction distances in this system appears to be weak. Figures 3e and 3f show that the interaction energy of FAD with the SWCNT at the primary pocket is almost equal to zero, while that interaction energy is still relatively small in the adenine region of FAD, being not larger than 0.4 kcal/mol. Therefore, it should prove to be quite difficult to affect conformational change in the FAD. The potential energy of isoalloxazine in system D is about 10 kcal/mol and is smaller than those in the other systems. Despite of the large bending deflection of FAD in system D, there is still very little variation in the potential fluctuation of FAD between all the systems, which may be attributed to the flexibility of the ribitol segment [22]. As shown in Figures 3c and 3d, the interaction energy of FAD with apo-GOx, which remains constant at ~ –400 kcal/mol, is similar for both systems B and C. Systems A and D have interaction energies of ~ –365 kcal/mol and ~ –340 kcal/mol, respectively. In terms of the conformational change of FAD shown in Figure 2 and the fluctuation of the interaction energy shown in Figs. 3c and 3d, it can be observed that the conformational fluctuation of FAD increases with a decrease in the interaction energy between FAD and apo-GOx, which implies that the interaction energy with apo-GOx is able to keep FAD stable.

This result also indicates that other forces regulate the mobility of FAD. As an important element of biochemical reactions, the water solution can also have a critical effect on the stability and activity of GOx. Through observing and comparing the molecular trajectories for the four different systems, the behaviour of water molecules appears to play a crucial role in the conformational change of FAD.

### 2.3. The interaction energy with water molecules

**Figure 4.**
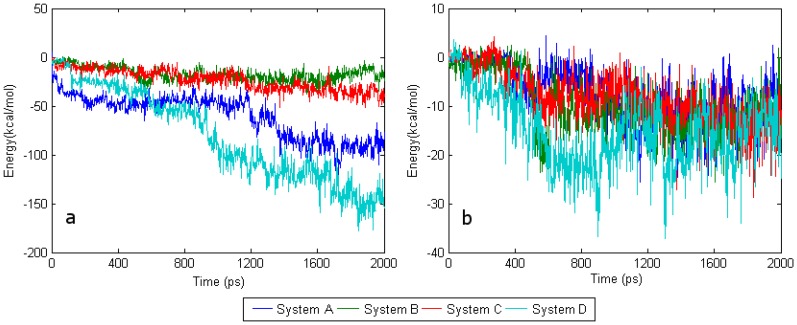
Trends for the interaction energy between FAD/isoalloxazine and water. (a) Interaction energy of FAD with water, (b) of isoalloxazine with water.

Figure 4 shows that the interaction energy between water molecules and FAD in systems A and D are larger than those in systems B and C. Taking the differences between the interaction energies with SWCNT and with water into consideration, shows that water molecules should play an essential role in the conformational change of FAD. Thorough analysis of the results shows that adsorption of the primary pocket on SWCNT decisively determines the energy state of the water molecules in the cavity. To some extent, the effect of SWCNT adsorption at the secondary pocket also determines by the energy state of the water molecules in the primary pocket. Comparing the interaction energy between FAD and water in system A with that in system D, the interaction energy in system A seems to be larger than that in system D during the first 600 ps. Conversely, the interaction energy in system D is smaller than that in system A after the first 600 ps. Subsequently, the interaction energies in both systems show a stepwise increment and reach a stable state at about 90 kcal/mol after 1.4 ns.

Figure 4 shows that between 300 ps and 500 ps the interaction energy between FAD and water in system A seems to be larger than that in system D. By contrast, the interaction energy between isoalloxazine and water in system D seems to be larger than that in system A. From these results, it is apparent that the interaction energy of the adenine terminal of FAD in system D should be far smaller than that in system A. Thus, the energy deviation between the isoalloxazine terminal and the adenine terminal in system D should be far larger than that in system A. Figure 3 shows that the interaction energy between FAD and apo-GOx decreases with compression deformation from the isoalloxazine terminal to the adenine terminal, indicating that the interaction energy of FAD with apo-GOx and that with water together could determine the tendency for the conformational change of FAD. At this point, the key role of the driving force from water was further clarified.

After ~600 ps, the interaction between FAD and water increase while that of isoalloxazine does not. The molecular trajectory analysis reveals that water begins to access the space between apo-GOx and FAD, so that the ribitol segment of FAD interacts with water, indicating that the effect of the interaction energy of ribitol with water may counterbalance the effect of that with isoalloxazine. As a result, the bending deflection of FAD starts to decline stepwise.

### 2.4. Conformational changes and the effect of water

Due to the different adsorption orientations and states of the SWCNT related to different adsorption sites, it should be evident that the significant difference in the dynamic processes of conformational change of FAD can be attributed to the impact of different activities and distributions of water.

Comparing the conformation-time curves and the time curves of the interaction energy with water given in the previous section for system A with those for systems B and C indicates that the activity and distribution of water in the vicinity of the primary pocket in systems B and C could be influenced by the adsorption on the SWCNT, resulting a low water density and a weaker activity. In systems B and C, the interaction energy of FAD with the catalytic site is less due to the weak activity of water. By contrast, due to the openness of the substrate channel to the catalytic site, the action and distribution of water in system D appears to be similar to that in system A. System D it is even more active than system A with SWCNT adsorbing at the secondary pocket.

It seems reasonable that the accessibility of the channel to the primary pocket may be controlled by the different strengths of the hydrophobic force of the SWCNT at different adsorption positions and orientations. In addition, direction observation of the molecular trajectories reveal that the SWCNT is not adsorbed near the entrance of the primary pocket in systems B and C. However, due to hydrophobic nature of the SWCNT, the activities of water in the vicinity of the primary pocket would eventually be significantly suppressed in those systems, and further block the free access of the catalytic substrate, β-D-glucose, to the catalytic center. Consequently, denaturation of GOx probably occurs in system C. This simulation result could make further understanding for those experimental results: the steric hindrance arising from the association of FAD with the apo-GOx component of the enzyme [13].

## 3. Experimental Section

GOx consists of apo-GOx and an embedded FAD coenzyme. It is a homodimer with a molecular weight of 150-180 kDa. The formula of FAD is shown in Figure 1e. The X-ray structure coordinates of *Aspergillus niger* GOx (PDB entry 1GAL) (EC 1.1.3.4) was refined to a resolution of 2.3 Å in ref. [21]. A monomer from the GOx homodimer was used in this study. SWCNTs with (7, 7) armchair chiralities were generated using the program Tubegen [23]. Because of representing the inherent characteristics of high surface area, the sidewall of SWCNT was taken as the important sites to be investigated.

There are two pockets closer to the FAD coenzyme than other surface sites of GOx. In this paper, the one close to isoalloxazine is denoted as the primary pocket since it is where the catalysis process occurs; the other pocket is close to the adenine of FAD and it is referred to as the secondary pocket since it is regarded as the auxiliary channel for relay electrons generated at the catalysis active site [2,28]. The shorter distance of the redox active cofactor of the GOx can make more profit of direct electron transfer by tunnelling effect. So far, these two pockets were used as key sites for building three different adsorption orientations to examine the effect of the FAD coenzyme that is influenced by the interaction between GOx and SWCNTs on the conformational mobility. Other positions on the surface of GOx were not considered due to their distance from the catalytic sites [13]. In addition, a system of GOx without SWCNTs was generated and denoted as system A in order to compare the effect of absorption on the sidewalls of the SWCNTs. The other three systems were generated and denoted as systems B, C and D based on the location, state and orientation of FAD in apo-GOx. All four systems are shown in Figure 1a.

Simulations were performed in the following manner. First, 100 energy minimization iterations were performed, followed by equilibration of system A in an aqueous solution. A SWCNT was generated with a length of 97.2 Å. It was sufficiently long so that any influence of the edge effect for the adsorption of GOx on SWCNTs with different starting orientations could be ignored. This was done to take the effects of the periodical conditions and the dimensions of GOx into consideration. The 200-ps equilibrated GOx without SWCNTs was used as the initial state for systems B, C and D. Then, 100 iterations of energy minimization were performed for all three systems.

All four systems were solvated in a box of TIP3 water. The water box should be large enough to contain all the systems. All systems then underwent 2 ns MD runs. All MD simulations were performed using the program NAMD [24]. The force field parameters for the SWCNT and the apo-GOx were taken from CHARMM27 [25], and the force field of the cofactor FAD was empirically constructed from a combination of FMN and ATP in GROMACS [22]. The parameters for the Lennard–Jones potential for the cross interactions between non-bonded atoms were obtained from the Lorentz–Berthelot combining rule [26]. All the geometric structures of the different systems were visualized by VMD [27]. All simulations were performed with a time step of 2 fs, and a cut-off was set with a switching function starting at a distance 10 Å and reaching zero at 12 Å. A particle mesh Ewald (PME) summation was used to calculate the long-range electrostatic interactions, with a cut-off distance of 12 Å for the separation of the direct and reciprocal space. During the MD simulations, the Langevin method to ensure a constant temperature of 310 K and a constant pressure of 101.3 kPa. Periodic boundary conditions were applied for all the simulations. The interaction and potential energies were calculated and analyzed using the tools included in the VMD environment.

## 4. Conclusions

Molecular dynamic simulations for the interaction between glucose oxidase and SWCNT at three different positions on the catalytic surface were performed to investigate the dynamic mechanism of conformation of the ensemble at the catalytic site. The results demonstrate that the MD simulation can provide insight into the changes in the nanostructure and interaction processes between SWCNT and the protein. The following detailed conclusions can be drawn:

The conformational mobility of ensemble of FAD coenzyme differs distinctly depending on its adsorption position on the catalytic surface. The driving force of conformational change of the FAD coenzyme originates from the direct interaction energy with water, but not from that with SWCNT. The effect of the interaction with SWCNT on conformational change could be attributed to the change in the water distribution caused by the hydrophobicity of the SWNCT. In addition, the change in the water distribution and the reduction in the transferability of water in the vicinity of the catalytic site could block the free access of β-D-glucose to the catalytic site. Thus, the adsorption of the primary pocket site on the SWCNT could be another factor that causes denaturing of the catalyst.

## References

[1] Wilson R., Turner A.P.F. (1992). Glucose oxidase: an ideal enzyme. Biosens. Bioelectron..

[2] Xiao Y., Patolsky F., Katz E., Hainfeld J.F., Willner I. (2003). “Plugging into enzymes”: nanowiring ofredoxenzymes by a gold nanoparticle. Science.

[3] Ramanavicius A., Kausaite A., Ramanaviciene A. (2005). Biofuel cell based on direct bioelectrocatalysis. Biosens. Bioelectron..

[4] Ivnitski D., Branch B., Atanassov P., Apblett C. (2006). Glucose oxidase anode for biofuel cell based on direct electron transfer. Electrochem. Commun..

[5] Rivas G.A., Rubianes M.D., Rodríguez M.C., Ferreyra N.F., Luque G.L., Pedano M.L., Miscoria S.A., Parrado C. (2007). Carbon nanotubes for electrochemical biosensing. Talanta.

[6] Davis F., Higson S.P.J. (2007). Biofuel cells-Recent advances and applications. Biosens. Bioelectron..

[7] Frew J.E., Hill H.A.O. (1988). Direct and indirect electron transfer between electrodes and redox proteins. Eur. J. Biochem..

[8] Hess C.R., Juda G.A., Dooley D.M., Amii R.N., Hill M.G., Winkler J.R., Gray H.B. (2003). Gold electrodes wired for coupling with the deeply buried active site of arthrobacter globiformis amine oxidase. J. Am. Chem. Soc..

[9] Aguey-Zinsou K.F., Bernhardt P.V., Kappler U., McEwan A.G. (2003). Direct electrochemistry of a bacterial sulfite dehydrogenase. J. Am. Chem. Soc..

[10] Lyons M.E.G., Keeley G.P. (2006). The redox behaviour of randomly dispersed single walled carbon nanotubes both in the absence and in the presence of adsorbed glucose oxidase. Sensors.

[11] Iijima S. (1991). Helical microtubules of graphitic carbon. Nature.

[12] Balasubramanian K., Burghard M. (2006). Biosensors based on carbon nanotubes. Anal. Bioanal. Chem..

[13] Guiseppi-Elie A., Lei C., Baughman R.H. (2002). Direct electron transfer of glucose oxidase on carbon nanotubes. Nanotechnology.

[14] Cai C., Chen J. (2004). Direct electron transfer of glucose oxidase promoted by carbon nanotubes. Anal. Biochem..

[15] Tatke S.S., Renugopalakrishnan V., Prabhakaran M. (2004). Interfacing biological macromolecules with carbon nanotubes and silicon surfaces: a computer modelling and dynamic simulation study. Nanotechnology.

[16] Shen J.W., Wu T., Wang Q., Kang Y. (2008). Induced stepwise conformational change of human serum albumin on carbon nanotube surfaces. Biomaterials.

[17] Shen J.W., Wu T., Wang Q., Pan H.H. (2008). Molecular simulation of protein adsorption and desorption on hydroxyapatite surfaces. Biomaterials.

[18] Chen X., Wu T., Wang Q., Shen J.W. (2008). Shield effect of silicate on adsorption of protein onto silicon-doped hydroxyapatite (100) surface. Biomaterials.

[19] Zhou H., Wu T., Dong X., Wang Q., Shen J.W. (2007). Adsorption mechanism of BMP-7 on hydroxyapatite (001) surfaces. Biochem. Biophys. Res. Commun..

[20] Lin C.S., Zhang R.Q., Niehaus T.A., Frauenheim T. (2007). Geometric and electronic structures of carbon nanotubes adsorbed with flavin adenine: a theoretical study. J. Phys. Chem. C.

[21] Hecht H.J., Kalisz H.M., Hendle J., Schmid R.D., Schomburg D. (1993). Crystal structure of glucose oxidase from Aspergillus niger refined at 2.3 Å resolution. J. Mol. Biol..

[22] van den Berg P.A.W., Feenstra K.A., Mark A.E., Berendsen J.C., Visser J.W.G. (2002). Dynamic conformations of flavin adenine dinucleotide: simulated molecular dynamics of the flavin cofactor related to the time-resolved fluorescence characteristics. J. Phys. Chem. B.

[23] Frey J.T., Doren D.J. (2003). TubeGen..

[24] Phillips J.C., Braun R., Wang W., Gumbart J., Tajkhorshid E., Villa E., Chipot C., Skeel R.D., Kalé L., Schulten K. (2005). Scalable molecular dynamics with NAMD. J. Comput. Chem..

[25] MacKerell A.D., Bashford D., Bellott M., Dunbrack R.L., Evanseck J.D., Field M.J., Fischer S., Gao J., Guo H., Ha S., Joseph-McCarthy D., Kuchnir L., Kuczera K., Lau F.T.K., Mattos C., Michnick S., Ngo T., Nguyen D.T., Prodhom B., Reiher W.E., Roux B., Schlenkrich M., Smith J.C., Stote R., Straub J., Watanabe M., Wiórkiewicz-Kuczera J., Yin D., Karplus M. (1998). All-atom empirical potential for molecular modeling and dynamics studies of proteins. J. Phys. Chem. B.

[26] Hirschfelder J.O., Curtiss C.F., Brid R.B. (1954). Molecular theory of gases and liquids.

[27] Humphrey W., Dalke A., Schulten K. (1996). VMD: visual molecular dynamics. J. Mol. Graphics.

[28] Patolsky F., Weizmann Y., Willner I. (2004). Long-range electrical contacting of redox enzymes by SWCNT connectors. Angew. Chem. Int. Ed..

[29] Wohlfahrt G., Trivic S., Zeremski J., Pericin D., Leskovac V. (2004). The chemical mechanism of action of glucose oxidase from Aspergillus niger. Mol. Cell. Biochem..

